# The Effects of Two Servings of a Thermogenic Supplement on Metabolism, Hemodynamic Variables, and Mood State Outcomes in Young Overweight Adults

**DOI:** 10.7759/cureus.54484

**Published:** 2024-02-19

**Authors:** Christine M Florez, Jessica Prather, Dylon Miller, Amie Vargas, Bella Soto, Abby Harrison, Grant Tinsley, Lem Taylor

**Affiliations:** 1 Human Performance Lab, University of Mary Hardin-Baylor, Belton, USA; 2 Kinesiology & Sport Management, Texas Tech University, Lubbock, USA; 3 Physiology and Nutrition, University of Mary Hardin-Baylor, Belton, USA

**Keywords:** body composition, basal metabolic rate, supplement, pre-workout supplement, nutrition and metabolism

## Abstract

Introduction

We examined if acute ingestion of a novel thermogenic supplement influences resting energy expenditure (REE), mood, and hemodynamic function.

Methods

Forty-six adults completed this randomized, placebo-controlled, double-blind, crossover study. Participants underwent two conditions: placebo (PL) and treatment (TX) containing 300 mg of caffeine and 3 g of acetyl-L-carnitine. REE, systolic blood pressure (SBP), diastolic blood pressure (DBP), heart rate (HR), and mood states were assessed at baseline and 30, 60, and 120 minutes post-ingestion. Data were analyzed using repeated measures analysis of variance.

Results

A significant condition-by-time interaction was observed for REE. At the 30-, 60-, and 120-minute post-ingestion timepoints, REE was 202 ± 26, 238 ± 40, and 209 ± 29 kcal/d greater in the TX condition compared to PL. No significant differences were observed for SBP and HR but a significant interaction indicated that DBP was elevated at 30 minutes in the TX vs. PL, though values remained within normal ranges. Significant interactions were observed for perceived alertness, concentration, energy, and focus, with increases in TX.

Conclusion

These data provide evidence that acute consumption of the thermogenic dietary supplement OxyShred (EHPlabs, Salt Lake City, Utah, USA) stimulates increases in REE that are sustained for ≥ two hours, along with increasing perceived alertness, concentration, energy, and focus. Changes in hemodynamic function are minimal and within normal ranges.

## Introduction

The obesity epidemic has led to increased interest in exogenous aids for the improvement of body composition. Thermogenic aids, also referred to as “fat burners,” are a category of weight loss supplements commercially available to consumers. The popularity of thermogenic aids continues to rise with claims that their ingestion may influence resting energy expenditure (REE) and facilitate fat loss. Current evidence supports an acute increase in REE following the ingestion of a thermogenic supplement, although the magnitude of its effect may be influenced by the included ingredients and dose [1]. Thermogenic aids typically include a blend of ingredients thought to augment metabolic function. The blend of active ingredients is dependent on the manufacturer and is in many cases proprietary, leading to gaps in available literature regarding their effect on safety, metabolism, and subjective outcomes; however, the main ingredient in thermogenic supplements is often caffeine.

Caffeine is a well-studied sympathomimetic known to increase REE [2]. Due to its sympathomimetic properties, caffeine possesses the ability to stimulate lipolysis and affect metabolism through differing mechanisms. Caffeine acts as a competitive inhibitor for adenosine, a neurotransmitter that initiates a “drowsy” sensation [3]. Binding to adenosine α1 and α2A receptors is the primary action through which caffeine affects metabolism, indirectly activating the sympathetic nervous system (SNS) by allowing catecholamines to circulate longer. This activation of the SNS is associated with increased energy, suppressed appetite, and increased REE [4]. The secondary action by which caffeine influences lipolysis is through the inhibition of phosphodiesterase (PDE). Preventing PDE from hydrolyzing cyclic adenosine monophosphate (cAMP) to adenosine monophosphate (AMP) leads to increased cAMP accumulation, ultimately facilitating the activation of the hormone-sensitive lipase (HSL), the enzyme responsible for hydrolyzing triacylglycerols in the process of lipolysis.

The myriad of additional ingredients used in combination with caffeine to promote lipolysis include acetyl-L-carnitine (ALC), conjugated linoleic acid (CLA), and/or p-synephrine. Endogenous L-carnitine transports free fatty acids into the mitochondria for use as energy during beta-oxidation. Exogenous supplementation has been shown to increase muscle concentrations of L-carnitine with evidence to suggest that ALC supplementation may improve exercise performance [5]. Conjugated linoleic acid refers to a group of fatty acids found mainly in ruminant meat. Despite conflicting data, chronic CLA supplementation has been purported to promote body composition improvements [6,7]. Finally, the active ingredient found in bitter orange extract is called p-synephrine. Like caffeine, it elicits a sympathomimetic influence; however, a key difference is its target receptor. Caffeine targets the α1 adenosine receptors located in vascular smooth muscle and α2A receptors located in the brain. P-synephrine binds to adenosine receptor α3 and bears no influence on cardiovascular function [8].

The acute effects of multi-ingredient thermogenic supplements on REE have been explored in several experimental designs, with the majority observing significant increases in REE when compared to a placebo condition [9-17]. Study design, approaches to supplementation (dosages, form administration, etc.), and the ingredients within the proprietary blends of thermogenic supplements all vary, which makes consensus on the magnitude of their efficacy inconclusive.

In a previous study, our lab showed an increase in REE with a single dose of a multi-ingredient, thermogenic supplement consisting of 150 mg of caffeine [17]. Many thermogenic agents recommend one or two servings depending on the individual’s tolerance [18]. Additionally, commercially available pre-workouts and thermogenic aids include doses greater than 150 mg [19]. Therefore, the purpose of this study was to determine the effect of two servings of a commercially available multi-ingredient, thermogenic supplement containing 300 mg of caffeine, CLA, p-synephrine, and 3 g of ALC on REE, heart rate, blood pressure, and subjective outcomes.

## Materials and methods

Experimental design

There were two testing sessions in total separated by one week. Participants were instructed to avoid exercise and abstain from caffeine for at least 24 hours before testing appointments and to report to each testing session in a fasted state of at least 10 hours. Additionally, participants were required to complete a nutritional intake log for 48 hours before appointments.

Upon arrival to the laboratory for testing session one, anthropometric measurements of height (Seca 264 Digital Stationary Stadiometer, Hamburg, Germany) and weight (Tanita Body Composition Analyzer, model TBF-310, Arlington Heights, Illinois, USA) were obtained and recorded. Supplementation order was then determined using the website random.org. Following randomization, pre-ingestion values for heart rate (HR), systolic blood pressure (SBP) and diastolic blood pressure (DBP) (BP Omron Professional Intellisense Blood Pressure Monitor, model HEM-907XL, Kyoto, Japan), REE and respiratory exchange ratio (RER) (ParvoMedics, Sandy, Utah, USA), hunger/satiety, and perceptions of concentration, focus, alertness, energy, and fatigue were collected and then repeated at 30, 60, and 120 minutes post-ingestion of assigned supplement. REE was measured using a canopy system while participants were positioned supine, in accordance with manufacturer recommendations. Each subjective outcome was reported using a Likert scale. Based upon methods in previously published research, perceptions of hunger and satiety were rated on a scale ranging from 0 to 10 while perceptions of concentration, focus, alertness, energy, and fatigue were each rated on a scale ranging from 0 to 5 [17]. All measured variables were collected while the participant was in a supine position after having rested for at least five minutes. An adverse side effects questionnaire was completed following the 120-minute timepoint to assess any discomfort with supplementation. The second testing session was completed seven days later in the exact same manner as testing session one with the alternative treatment.

Supplement administration

At each testing session, participants ingested two servings of a powdered form of their respective treatment mixed with 600 mL of room-temperature water until dissolved. The ingredients in the active treatment (OxyShred Thermogenic Fat Burner, EHPlabs, Salt Lake City, Utah, USA) are presented in Tables 1, 2, while the placebo contained only inactive ingredients (gum Arabic, citric acid, malic acid, natural (NAT) watermelon type, NAT bitter blocker, sucralose, silicon dioxide, calcium silicate, and beet color powder).

**Table 1:** The ingredient list The table displays the listed ingredients for two servings of a commercially available thermogenic, OxyShred. * Proprietary blend ingredients.

Treatment ingredients	Amount/serving
Total carbohydrate	2 g
Dietary fiber	0.4 g
Vitamin C	346 mg
Thiamin	1.12 mg
Riboflavin	1.56 mg
Niacin	40 mg
Vitamin B6	1.96 mg
Vitamin B12	1.8 mcg
Pantothenic acid	3.4 mg
Chromium picolinate	20 mcg
Fat-burning matrix*	4006 mg
Immunity booster and prebiotic* complex	1250 mg
Mood enhancer matrix*	1702 mg
Full B vitamin spectrum*	49.18

**Table 2:** Proprietary blend ingredients The table displays specific ingredients included in each proprietary blend.

Proprietary blend (mg)	Ingredients
Fat-burning matrix (4006 mg)	Acetyl-L-carnitine HCl, Garcinia cambogia fruit extract (60% hydroxycitric acid), conjugated linoleic acid (CLA), grapefruit seed extract 4:1, raspberry ketones (from raspberry fruit extract), Mangifera indica seed extract, bitter orange fruit extract, green coffee bean extract (50% chlorogenic acid), olive leaf extract (10% oleuropein), guggul extract powder, chromium picolinate)
Immunity booster and prebiotic complex (1250 mg)	L-glutamine, inulin fiber, vitamin C (ascorbic acid)
Mood enhancer matrix (1702 mg)	L-tyrosine, taurine, caffeine anhydrous, Huperzia serrata whole herb extract (huperzine A)
Full B vitamin spectrum (49.18 mg)	Niacinamide (niacin), calcium pantothenate (pantothenic acid), pyridoxine HCl (vitamin B6), riboflavin (vitamin B2), thiamine mononitrate (vitamin B1), cyanocobalamin (vitamin B12)

Statistical analysis

Data were analyzed using R (v. 4.1.2; R Foundation for Statistical Computing, Vienna, Austria). Repeated measures analysis of variance (ANOVA) tests were performed using the *afex* R package [19] with condition (TX and PL) and time (pre and 30, 60, and 120 minutes post-ingestion) specified as within-subjects factors. The normality of residuals was examined by visual inspection of quantile-quantile plots, the Greenhouse-Geisser correction was used for violations of sphericity, and the *rstatix* R package [20] was used to identify outliers. Follow-up for significant effects was performed using pairwise comparisons with Tukey adjustment via the *emmeans* R package [21]. Dietary intake prior to each visit was compared using paired samples t-tests. The incidence of side effects between conditions was compared using paired Wilcoxon signed-rank tests. Statistical significance was accepted at p < 0.05.

## Results

Dietary intake

There were no observed differences in energy (kcal) or macronutrient intake between laboratory visits. Energy, carbohydrate, protein, and fat intake in the TX condition was (mean ± SD) 1777 ± 575 kcal, 182 ± 71 g, 92 ± 57 g, and 78 ± 52 g, respectively. In the PL condition, intake for energy, carbohydrate, protein, and fat was 1735 ± 628 kcal, 180 ± 72 g, 96 ± 50 g, and 69 ± 27 g, respectively.

Metabolism

Resting Energy Expenditure (REE)

**Figure 1:**
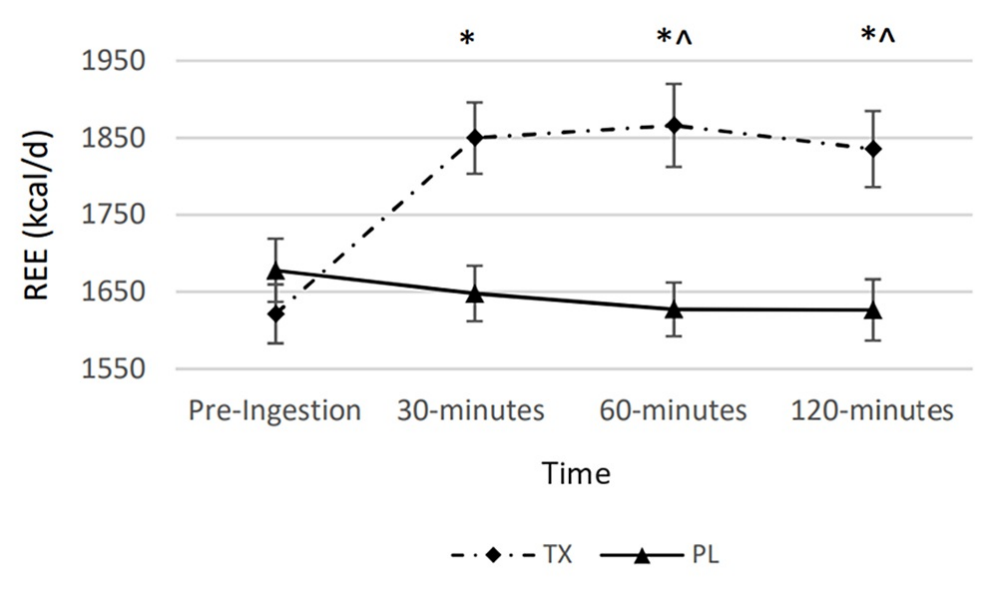
Changes in resting energy expenditure The graph displays changes in resting energy expenditure (REE) represented by kilocalories over the course of 120 minutes. Solid lines represent the placebo (PL) condition. Dashed lines represent the treatment (TX) condition. * = significantly greater than the “Pre” value in the treatment condition (p < 0.05); ^ = significantly greater than the corresponding value in the placebo condition (p < 0.05).

A statistically significant condition by time interaction was observed for REE (p < 0.001). Post hoc analysis revealed statistically significant differences between conditions at all post-ingestion timepoints. At the 30-, 60-, and 120-minute post-ingestion timepoints, REE was 202 ± 26 kcal/day, 238 ± 40 kcal/day, and 209 ± 29 kcal/day greater in the TX condition as compared to the PL condition, respectively. There were observed significant main effects for time and condition for REE (p < 0.001 for both). Results are displayed in Figure 1 and Table 3.

**Table 3:** Analysis of variance (ANOVA) results The table displays p-values and partial eta-squared values for measured variables. * Denotes statistical significance. REE = resting energy expenditure; RQ = respiratory quotient; HR = heart rate; SBP = systolic blood pressure; DBP = diastolic blood pressure.

Variable	Condition		Time		Condition x time	
	p-value	PES	p-value	PES	p-value	PES
REE	<0.0001*	0.53	<0.0001*	0.24	<0.0001*	0.43
RQ	0.34	0.02	<0.0001*	0.31	<0.0001*	0.19
HR	0.04*	0.09	<0.0001*	0.19	0.12	0.04
SBP	0.1	0.06	0.04*	0.06	0.06	0.06
DBP	0.005*	0.16	0.0006*	0.14	0.007*	0.09
Alertness	0.03*	0.1	<0.0001*	0.23	0.002*	0.12
Concentration	0.08	0.1	0.0007*	0.14	0.005*	0.1
Energy	0.003*	0.18	<0.0001*	0.28	0.0005*	0.13
Fatigue	0.007*	0.15	0.01*	0.09	0.27	0.03
Focus	0.04*	0.09	<0.0001*	0.18	0.003*	0.11

Respiratory Exchange Ratio (RER)

There was a statistically significant condition by time interaction in RER (p < 0.05). Post hoc analysis revealed that at the 30-minute post-ingestion timepoint, RER was greater in the TX condition (RER = 0.87 ± 0.01) as compared to the PL condition (RER = 0.84 ± 0.01). There was also a statistically significant time main effect (p < 0.05) whereby the 30-minute timepoint was greater than all other timepoints, including pre-ingestion.

Hemodynamic function

Heart Rate (HR)

A statistically significant main effect was observed in HR for both conditions (p < 0.05) and time (p < 0.01); however, there was no condition-by-time interaction. Post hoc analysis indicated that HR was lower in the TX condition as compared to the PL condition. Additionally, it was observed for the time main effect that the 30-minute post-ingestion timepoint was 4 ± 0.8 bpm lower than pre-ingestion. Changes between the TX condition and PL condition are displayed in Table 4, with p-values and partial eta-squared shown in Table 3.

**Table 4:** Changes in hemodynamic variables The table represents measured results for hemodynamic variables. Values are represented as mean ± SD. HR = heart rate; SBP = systolic blood pressure; DBP = diastolic blood pressure.

Variable	Condition	Baseline	30 minutes	60 minutes	120 minutes
HR	TX	62.0±1.52	56.3±1.45	59±1.34	60.4±1.26
	PL	62.4±1.63	60.2±1.61	62.1±1.74	62.0±1.64
SBP	TX	113.8±1.69	119.2±2.22	117.6±1.7	117.5±1.88
	PL	114.7±1.74	114.6±1.71	115.6±1.61	116±1.82
DBP	TX	67.4±1.27	73.9±1.38	71.5±1.08	70.9±1.30
	PL	67.8±1.08	68.5±1.22	70±1.25	68.9±1.18

Systolic Blood Pressure (SBP)

No condition-by-time interactions were observed. There was a statistically significant time main effect observed for SBP (p = 0.04) whereby SBP was higher at the 60-minute post-ingestion timepoint compared to pre-ingestion across conditions.

Diastolic Blood Pressure (DBP)

A statistically significant condition by time interaction was observed (p < 0.05) in DBP. Post hoc testing revealed that the 30-minute post-ingestion timepoint was greater in the TX condition as compared to the PL condition. Moreover, statistically significant time and condition main effects were observed. In the TX condition, the 30- and 60-minute post-ingestion timepoints were greater than the pre-ingestion timepoint.

Subjective measures

Alertness

**Figure 2:**
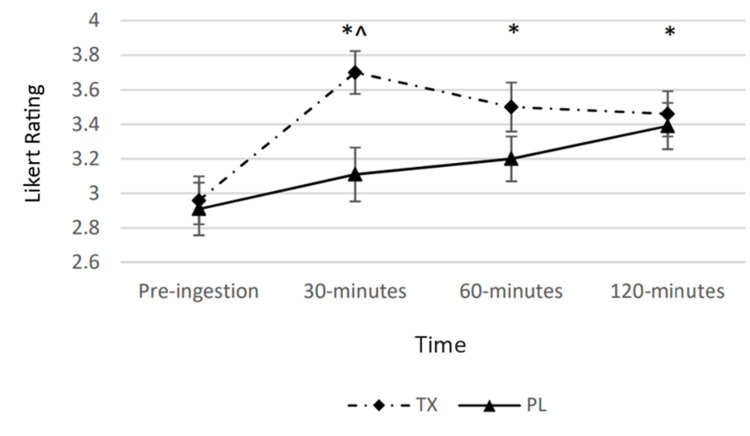
Changes in perceived alertness The graph displays changes in perceived alertness over the course of 120 minutes. Alertness was determined via a Likert scale ranging from 0 to 5 and measured at four timepoints over the course of 120 minutes. The solid line represents the placebo (PL) condition. The dashed line represents the treatment (TX) condition. * = significantly greater than the “Pre” value in the TX condition (p < 0.05); ^ = significantly greater than the corresponding value in the PL condition (p < 0.05).

There was a statistically significant condition by time interaction (p < 0.05) observed for perceived alertness. The main effects for time and condition were also revealed (p < 0.05). Post hoc analysis revealed that perceived alertness was greater in the TX condition at the 30-minute post-ingestion as compared to the PL condition. Moreover, 30-, 60-, and 120-minute post-ingestion timepoints in the TX condition were significantly greater than the pre-ingestion value (Figure 2).

Concentration

**Figure 3:**
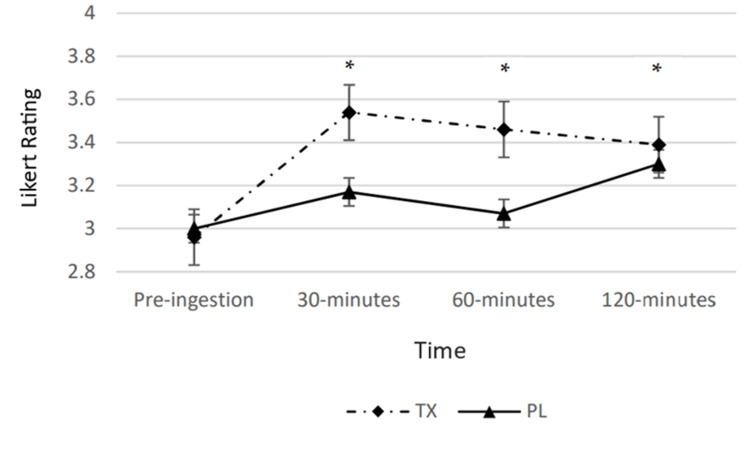
Changes in perceived concentration The graph displays changes in perceived concentration over the course of 120 minutes. Concentration was determined via a Likert scale ranging from 0 to 5 and measured at four timepoints over the course of 120 minutes. The solid line represents the placebo (PL) condition. The dashed line represents the treatment (TX) condition. * = significantly greater than the “Pre” value in the TX condition (p < 0.05); ^ = significantly greater than the corresponding value in the PL condition (p < 0.05).

A statistically significant condition-by-time interaction was observed for perceived concentration (p < 0.05). There was a statistically significant time main effect (p < 0.05); however, no condition main effects were observed. Post hoc analysis revealed that perceived concentration in the TX condition was significantly greater than pre-ingestion values at the 30-, 60-, and 120-minute post-ingestion timepoint (Figure 3).

Energy

**Figure 4:**
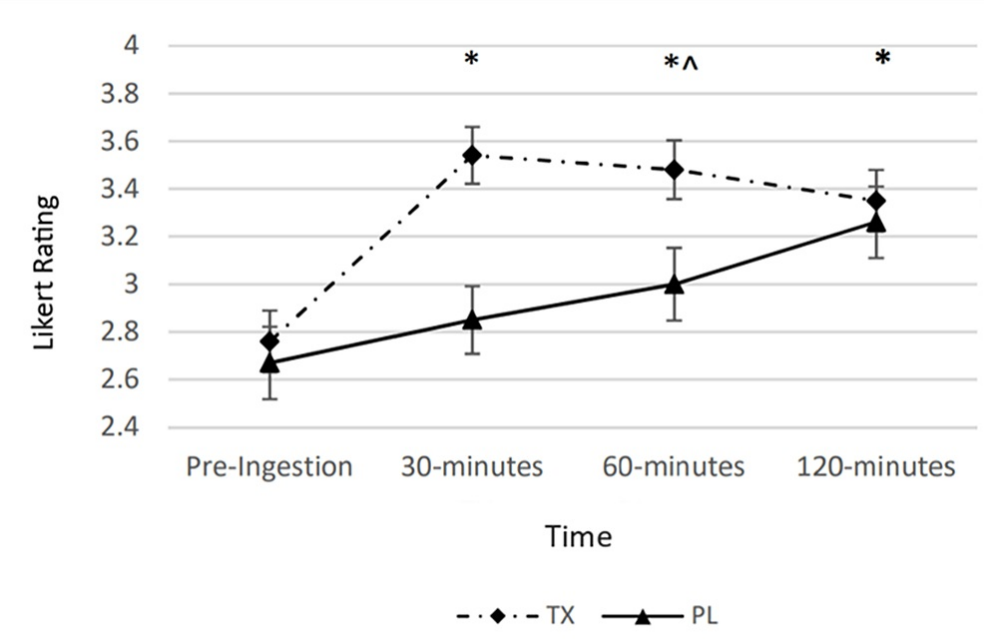
Changes in perceived energy The graph displays changes in perceived energy over the course of 120 minutes. Energy was determined via a Likert scale ranging from 0 to 5 and measured at four timepoints over the course of 120 minutes. The solid line represents the placebo (PL) condition. The dashed line represents the treatment (TX) condition. * = significantly greater than the “Pre” value in the TX condition (p < 0.05); ^ = significantly greater than the corresponding value in the PL condition (p < 0.05).

A statistically significant condition by time interaction was observed for perceived energy (p < 0.05). There was both a statistically significant condition main effect and time effect (p < 0.05). Post hoc analysis revealed that perceived energy in the TX condition was significantly greater than the PL condition at the 30- and 60-minute post-ingestion timepoint as compared to the PL condition. Additionally, perceived energy in the TX condition at the 30-, 60-, and 120-minute post-ingestion timepoints was significantly greater than the pre-ingestion value (Figure 4).

Fatigue

There was no condition-by-time interaction observed for perceived fatigue (p = 0.27); however, there were both time and condition main effects observed (p < 0.05). Post hoc analysis revealed that across conditions, the 120-minute post-ingestion timepoint was significantly lower than the pre-ingestion perceived fatigue value. Results are displayed in Table 2.

Focus

**Figure 5:**
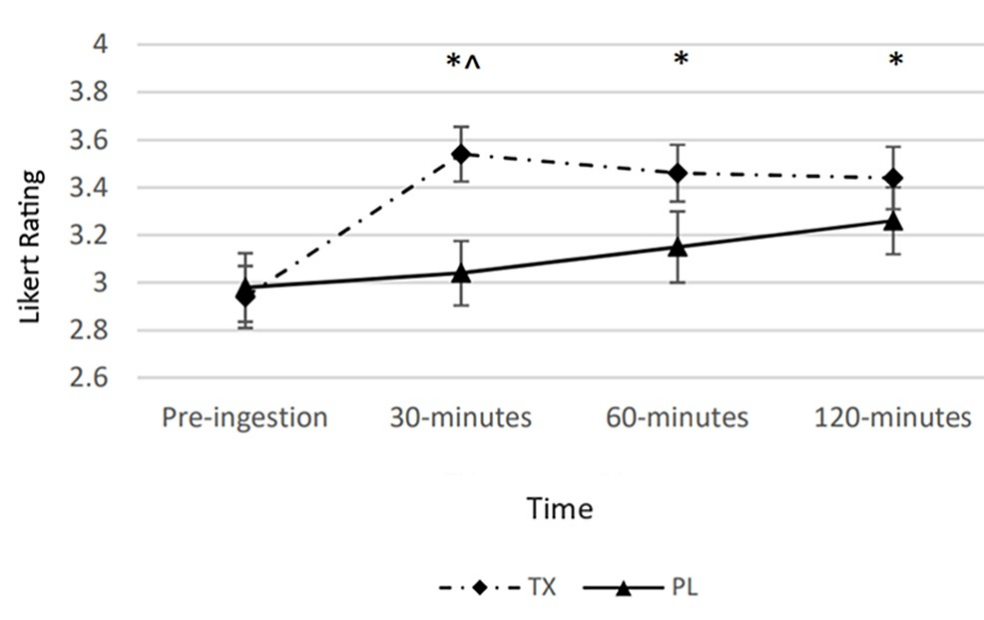
Changes in perceived focus The graph displays changes in perceived focus over the course of 120 minutes. Focus was determined via a Likert scale ranging from 0 to 5 and measured at four timepoints over the course of 120 minutes. The solid line represents the placebo (PL) condition. The dashed line represents the treatment (TX) condition. * = significantly greater than the “Pre” value in the TX condition (p < 0.05); ^ = significantly greater than the corresponding value in the PL condition (p < 0.05).

A statistically significant condition-by-time interaction was observed in perceived focus (p < 0.05). Additionally, a time main effect and a condition main effect were observed (p < 0.05). Post hoc analysis revealed that perceived focus was significantly greater in the TX condition at the 30-minute post-ingestion timepoint as compared to the PL condition (Figure 5). Moreover, perceived focus in the TX condition was significantly greater at the 30-, 60-, and 120-minute post-ingestion timepoints when compared to the pre-ingestion value.

Hunger and Satiety

There was not an observed condition by time interaction for hunger and satiety. Main effects for time (p < 0.05) and condition (p < 0.05) were observed. Post hoc analysis revealed that perceived hunger and satiety were greater in the TX condition as compared to the PL.

Side Effects

There was no difference between groups for the occurrence of dizziness (p = 0.49), headache (p = 0.29), heart racing (p = 0.65), heart skipping (p = 1.0), shortness of breath (p = 0.48), nervousness (p = 0.57), or blurred vision (p = N/A; scores of zero for all participants).

## Discussion

Excessive storage of adipose tissue is associated with negative health outcomes, including metabolic syndrome and increased risk of cardiovascular events. Despite this correlation, the rate of obesity incidence continues to rise as does interest in its prevention and treatment. For individuals seeking to decrease body fat, a calories in vs. calories out (CICO) strategy is often advised for weight loss. Thermogenic supplements are a nutritional intervention used by consumers to increase caloric expenditure with the hope of contributing to overall fat loss. Current literature reports acutely elevated metabolism with the ingestion of a thermogenic supplement [9-18,22-25].

The purpose of this investigation was to determine the acute effects of two servings of a commercially available thermogenic supplement on REE, RER, hemodynamic variables, hunger/satiety, and the subjective perceptions of alertness, concentration, energy, fatigue, and focus. Primary findings of these data indicate that the acute ingestion of a thermogenic dietary supplement containing 300 mg of caffeine and a proprietary blend that includes CLA, ALC, and p-synephrine yields a greater metabolic response than placebo over the course of 120 minutes. Additionally, RER was greater at the 30-minute post-ingestion timepoint in the TX condition (RER = 0.87 ± 0.01) as compared to the PL condition (RER = 0.84 ± 0.01). Although no observable differences were seen in HR and SBP, DBP in the TX condition peaked at the 30-minute post-ingestion point at 73.9 mmHg and was significantly greater than in the PL condition by 5.4 mmHg. Despite the elevated DBP measured in the TX condition, the value remained within an acceptable reference range. Moreover, perceptions of alertness, concentration, and focus in the TX condition were greater than in the PL condition. These findings suggest that supplementation with this thermogenic blend acutely elevates metabolism and increases subjective outcomes when compared to a placebo. Finally, there were no reported adverse side effects such as dizziness, rapid HR, shortness of breath, and nervousness.

The observed findings reported here are in accordance with established literature whereby ingestion of a thermogenic supplement acutely increases REE [9-17,22-24]. Taylor and colleagues [23] observed REE increases up to three hours following the ingestion of a thermogenic supplement containing 400 mg of caffeine with metabolic changes of about 14%. A similar finding was observed in a later study investigating a 400 mg caffeine dose in tablet form [24]. Ziegenfuss and colleagues [25] conducted an experimental design that assessed the effects of a different thermogenic supplement and recorded increases in REE ranging from 15% to 19%. Assessing the metabolic effects of a thermogenic supplement containing 200 mg of caffeine, Campbell and colleagues [1] observed a significant shift in metabolism with increases ranging from 7% to 9%.

There is clear evidence that metabolic rate acutely increases with the ingestion of a thermogenic supplement; however, there is a lack of consistency in defining the magnitude of metabolic change elicited by supplementation. The range of reported changes in similar studies may be caused by the active ingredients within the proprietary blends as well as a range of caffeine dosing. Finally, individual differences amongst participants may also contribute to variation in estimated metabolic rate, for example, genotypes that encode enzymes responsible for caffeine metabolism [26,27], initial caffeine habituation, and body composition.

In the current investigation, the percent changes of REE observed in the TX condition ranged from 13% to 15% when comparing pre-ingestion and post-ingestion timepoints. At its peak, which was observed at the 60-minute timepoint, REE measured in the TX condition was 238 ± 26 kcal/d greater than in the PL condition. Moreover, in the final measured timepoint, REE in the TX condition remained elevated by 209 ± 29 kcal/d in comparison to the PL condition. The relationship between energy intake and expenditure is a primary influence on decreases in body mass. Therefore, determining the entire duration of REE elevation elicited by this thermogenic supplement and to what degree it contributes to overall total daily energy expenditure (TDEE) should be investigated.

The sympathomimetic properties of caffeine and its influence on increasing metabolism are well established; thus, its inclusion in thermogenic supplements and subsequent metabolic increases with ingestion can in part be attributed to the actions of caffeine. Adenosine is an inhibitory neuromodulator of central nervous system (CNS) activity and a second messenger for a variety of chemical processes. Current research supports adenosine’s role in sleep, which translates to an increased perception of drowsiness [28]. Caffeine is an adenosine antagonist that binds to α1 and α2a receptors allowing for increased circulation of catecholamines, which then bind to β2-adrenergic receptors located on adipocytes, thereby eliciting lipid mobilization through the activation of HSL [29]. Several acute studies have observed this effect with the ingestion of caffeine alone in varying doses [30-33]. In the present study, a caffeine dose of 300 mg seems to be the primary driver for increased metabolism over the course of two hours; however, caffeine is not the only ingredient with the capacity to influence REE.

Indeed, caffeine is often combined with additional active ingredients thought to maximize beta-oxidation. P-synephrine, ALC, and CLA are all ingredients proposed to aid in these processes and were present in the TX supplement of this study. P-synephrine is a thermogenic substance that has been shown to increase REE alone and in combination with caffeine without adverse side effects [33] while having no effect on cardiovascular function [7]. Conjugated linoleic acid is a fatty acid that is purported to inhibit lipogenesis as has been observed in some animal models [34]; however, human studies have produced differing results indicating that the benefits of CLA supplementation should continue to be investigated [35,36]. ALC is an ester of L-carnitine and is involved in the transport of fatty acids into the mitochondria from the cytosol for beta-oxidation [37].

The available literature regarding the benefits of ALC regarding metabolism and fat loss suggests that supplementation can upregulate pathways associated with insulin signaling and fatty acid metabolism [38]. Chronic supplementation and its effect on body composition are less conclusive with observations ranging from no change to a measurable improvement [39,40]. In the present study, the observed increase in REE without clear differences in RER - except for a transient between-condition difference 30 minutes following supplement ingestion - may indicate increases in both fat and carbohydrate oxidation following ingestion of the thermogenic supplement without a clear shift toward fat oxidation. The inclusion of blood variables to monitor potential fat mobilization would provide better insight into the influence of thermogenic supplementation on lipolysis beyond increased REE.

Hemodynamic variables measured in this investigation included SBP, DBP, and HR monitored at each timepoint. Though the TX condition experienced no observable changes in SBP, DBP increased when compared to the PL condition at the 30-minute timepoint. Additionally, the 30- and 60-minute post-ingestion timepoints were significantly greater than pre-ingestion values. Considering that activation of the SNS elicits vasoconstriction, the collective response to a 300 mg dose of caffeine may be the most logical explanation for increased DBP, an effect that was also observed in resistance-trained males by Campbell and colleagues [1]. It is worth noting that no detectable differences in SBP were present despite some studies reporting observed increases in SBP and DBP with the ingestion of a thermogenic supplement [11,14]. Moreover, the rise in DBP though significant, remained within normal limits. The HR response data are consistent with previous thermogenic research whereby no detectable shifts in HR were observed as compared to a placebo condition [8-13,22-24]. Finally, though caffeine ingestion is associated with an increased sympathetic response, when collapsed across timepoints, HR in the TX condition was lower than the PL condition (p = 0.04).

Subjective outcomes were analyzed while participants were at rest to determine the metabolic influence of the supplement alone. In the present investigation, the TX condition experienced increases in alertness, concentration, energy, and focus when compared to the PL condition. Perceived fatigue was lower in the treatment condition compared to the placebo condition when mean values were averaged over time. These results agree with findings recorded by Outlaw and colleagues [41], who observed a thermogenic supplement-induced increase in perceived alertness and focus. There is a high density of α2a receptors in the brain, and caffeine - a competitive inhibitor of adenosine - possesses the ability to cross the blood-brain barrier, which offers a possible explanation for the increase in these perceptions. However, the source and magnitude of this influence are yet to be clearly defined. In addition to perceptions of mental acuity, subjective reports of hunger and satiety levels were measured and observed to have no difference between conditions. These findings are consistent with those reported by Alkhatib and colleagues [13], who concluded that thermogenic supplementation has no influence on perceptions of hunger.

This study possesses three main limitations. First, the study design was acute in nature. The thermogenic supplement increased measured REE; however, it is unclear if this conclusion can be translated to chronic supplementation, specifically regarding metabolism and its potential to facilitate fat loss. Secondly, although this investigation included hemodynamic, metabolic, and subjective variables, no biochemical markers were analyzed. The inclusion of biochemical markers may better define the effects of thermogenic supplementation on beta-oxidation. Future investigations should employ a long-duration intervention and include measured biomarkers indicative of lipolysis. Moreover, because the current study looked at metabolism at rest, further investigation into thermogenic supplements versus placebo in conjunction with an exercise intervention would better define the benefits of supplementation on body composition changes. Finally, improved subjective outcomes were observed while participants were at rest. Improvements in perceived mental acuity due to the ingestion of a thermogenic supplement need to be researched more thoroughly to extrapolate its potential influence during the performance.

## Conclusions

The data from the present study suggest that the acute ingestion of a particular thermogenic supplement containing 300 mg of caffeine and 3 mg of ALC accelerates metabolic rate, resulting in an observed increase of REE for at least 120 minutes post-ingestion at rest. Though there was a transient increase in DBP, the values remained within normal limits. Furthermore, participants reported no adverse side effects such as nervousness, shortness of breath, headache, palpitations, blurred vision, and/or dizziness associated with the TX supplement. More research should be done with this supplement to confirm whether long-term use will contribute to elevated energy expenditure and weight loss. Additionally, a training program co-administered with this thermogenic supplement would better define the effectiveness of this product. The blend of ingredients in this thermogenic supplement can be explored further to better elucidate the response in populations with varying levels of adiposity.
